# Structure of the TPR Domain of AIP: Lack of Client Protein Interaction with the C-Terminal α-7 Helix of the TPR Domain of AIP Is Sufficient for Pituitary Adenoma Predisposition

**DOI:** 10.1371/journal.pone.0053339

**Published:** 2012-12-31

**Authors:** Rhodri M. L. Morgan, Laura C. Hernández-Ramírez, Giampaolo Trivellin, Lihong Zhou, S. Mark Roe, Márta Korbonits, Chrisostomos Prodromou

**Affiliations:** 1 Genome Damage and Stability Centre, University of Sussex, Brighton, United Kingdom; 2 Department of Endocrinology, Barts and the London School of Medicine, Queen Mary University of London, London, United Kingdom; 3 Biochemistry and Molecular Biology, Chichester 2, University of Sussex, Brighton, United Kingdom; Consiglio Nazionale delle Ricerche, Italy

## Abstract

Mutations of the *aryl hydrocarbon receptor interacting protein* (*AIP*) have been associated with familial isolated pituitary adenomas predisposing to young-onset acromegaly and gigantism. The precise tumorigenic mechanism is not well understood as AIP interacts with a large number of independent proteins as well as three chaperone systems, HSP90, HSP70 and TOMM20. We have determined the structure of the TPR domain of AIP at high resolution, which has allowed a detailed analysis of how disease-associated mutations impact on the structural integrity of the TPR domain. A subset of C-terminal α-7 helix (Cα-7h) mutations, R304* (nonsense mutation), R304Q, Q307* and R325Q, a known site for AhR and PDE4A5 client-protein interaction, occur beyond those that interact with the conserved MEEVD and EDDVE sequences of HSP90 and TOMM20. These C-terminal AIP mutations appear to only disrupt client-protein binding to the Cα-7h, while chaperone binding remains unaffected, suggesting that failure of client-protein interaction with the Cα-7h is sufficient to predispose to pituitary adenoma. We have also identified a molecular switch in the AIP TPR-domain that allows recognition of both the conserved HSP90 motif, MEEVD, and the equivalent sequence (EDDVE) of TOMM20.

## Introduction

**Figure 1:**
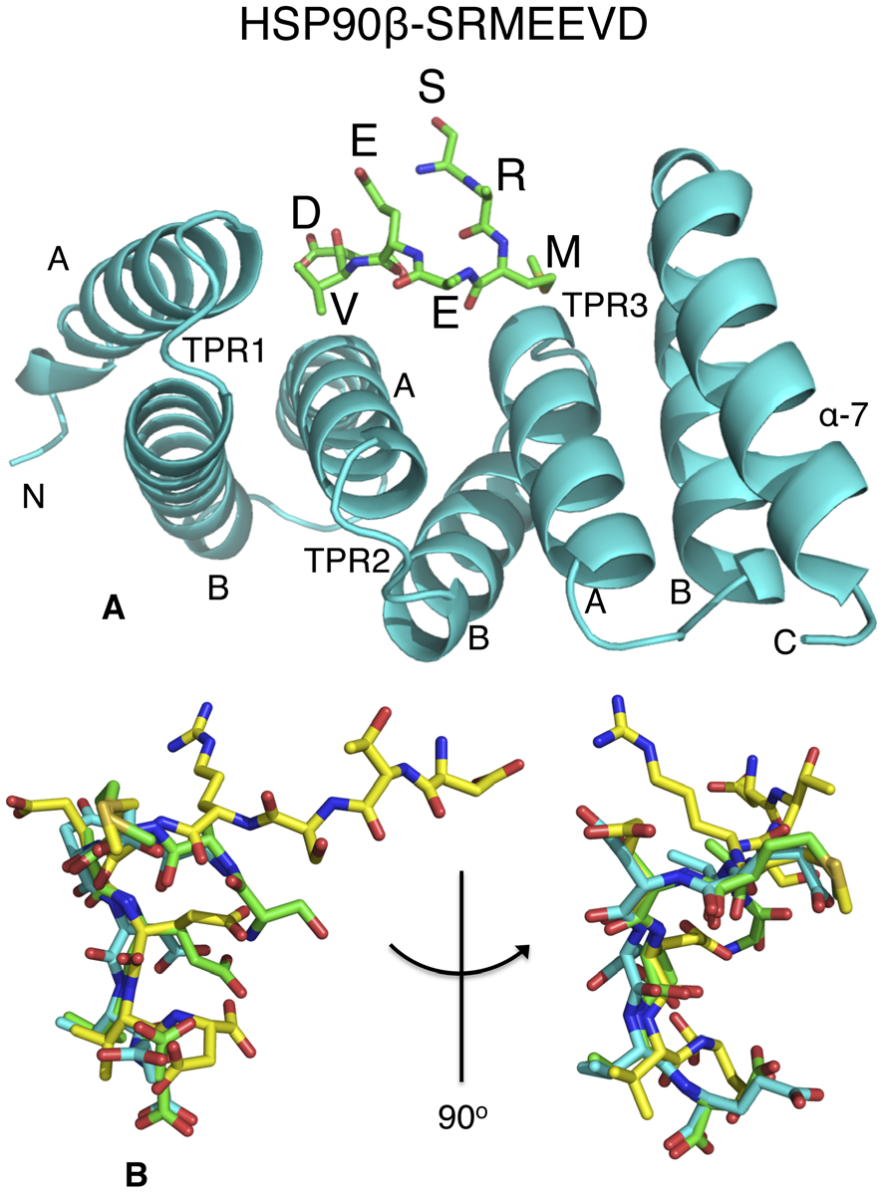
PyMol cartoon of the structure of human AIP. (A), PyMol cartoon of the HSP90β EDASRMEEVD-peptide (green) bound to the TPR domain of AIP (cyan). Only SRMEEVD of the peptide was visible. The structure was obtained at 2.0 Å (PDB, 4AIF) while that with the TOMM20 AQSLAEDDVE-peptide was obtained at 1.9 Å (PDB, 4APO, not shown). The A and B helices of each TPR motif (TPR1 to 3) and the C-terminal alpha helix (α-7) are indicated. (B), Superimposition of peptide conformations of HSP90β EDASRMEEVD (green), TOMM20 AQSLAEDDVE (cyan) bound to AIP (only SRMEEVD and AEDDVE of the peptides is shown), and HSP90α DTSRMEEVD (yellow) peptide bound to CHIP, showing that the peptide backbone conformation is essentially the same.

Recently, mutations in aryl hydrocarbon receptor interacting protein (AIP) [1], [2] have been linked to familial isolated pituitary adenomas (FIPA) [3]–[5], a condition most often characterized by young-onset growth hormone and prolactin-secreting pituitary tumors (reviewed by [6]), which leads to acromegaly and gigantism. The human *AIP* gene encodes a 37 kDa protein of 330 amino acids that, based on similarities to other proteins, is predicted to have an N-terminal immunophilin-like domain [7] and a C-terminal tetratricopeptide repeat (TPR) domain. Typically, TPR domains consist of three sets of a highly degenerate consensus sequence of 34 amino acids, often arranged in tandem repeats, formed by two alpha-helices forming an antiparallel amphipathic structure and a final C-terminal α-7 helix (Cα-7h; Fig. 1A). The TPR domain of AIP appears to be similar to the corresponding domains of HOP, CHIP, CYP40, PP5, FKBP51 and FKBP52 and the aryl hydrocarbon receptor-interacting protein like 1 (AIPL1) (Fig. 1A). Although the immunophilin domain of AIP shows significant homology to equivalent domains of FKBP12 and FKBP52, AIP does not bind immunosuppressant drugs such as FK506 and rapamycin [2] and displays no PPIase activity [8], [9].

AIP has been reported to interact with a number of different proteins: chaperones (HSP90, HSP70, TOMM20), and client proteins including nuclear receptors (AhR, ERα), phosphodiesterase 4A5 (rat isoform of human PDE4A4) and PDE2A3, survivin, G proteins, RET and EBNA3 amongst others (see recent review by [10]). Interestingly, HSP90, HSP70 and TOMM20 share a common conserved C-terminal motif, EEVD (HSP90 and HSP70) and DDVE (TOMM20) that potentially act as the binding sites for the AIP TPR-domain [11], [12]. A similar motif, EELD, has been identified in PDE4A5 [13]. HSP90 is a molecular chaperone that is involved in the maturation of many signal transduction proteins ([14]–[16]), while HSP70 is a more generalised protein-folding chaperone [17], [18]. In contrast, TOMM20 acts as a receptor for unfolded proteins destined for translocation across the outer mitochondrial membrane [19]. Together, these chaperones are responsible for the activation and maturation of a vast array of other proteins.

AhR, a client protein of the HSP90-AIP complex, may function as a tumor suppressor that becomes silenced. [4], [5], [20]–[25], but its precise role in predisposition to pituitary adenoma is not well understood. AhR binds environmental dioxins, such as the non-metabolizable agonist 2,3,7,8 tetra-chlorodibenzo-ρ-dioxin (TCDD), which is known to promote tumorigenesis, but it is unclear whether this has a role in AIP-related tumorigenesis.

The cAMP pathway is important for somatotroph cell function and proliferation. As AIP interacts with phosphodiesterases (PDE4A5 and PDE2A), enzymes which degrade cAMP, this interaction may have an important role in AIP-related pituitary tumorigenesis. AIP has an opposite effect on PDE4A5 and PDE2A function [13], [26], [27] and very little data exist on the possible interaction with other PDEs. As there are over 52 different PDEs known, this aspect remains an important field of study.

Recently, AIP was shown to inhibit ERα transcriptional activity and AIP mutations lead to enhanced ERα transcriptional activity. Prolonged and a high-level exposure to estrogen is a known risk factor for developing a variety of tumors [28]–[31] including pituitary tumors [32], [33]. Furthermore, AIP has also been shown to upregulate PLAGL1 (also known as ZAC1), a zinc finger protein with apoptotic and cell cycle arrest activity [34], [35].

Around 75% of AIP mutations completely disrupt the C-terminal TPR domain and/or the Cα-7h [36]. The vast majority of the missense variants affect the two final TPR-motifs and the Cα-7h, both of which are involved in protein interactions. The client proteins AhR and PDE4A5 have been shown to bind to the Cα-7h part of the AIP molecule. How the lack of AIP or its dysfunction leads to tumorigenesis and how interactions are disrupted that predispose cells to tumorigenesis are poorly understood and difficult to predict as AIP interacts directly with a number of proteins and indirectly, via the three chaperone systems, with a bewildering number of proteins [10].

Here we aim to classify the effect of a variety of FIPA associated mutations on the structural integrity of AIP. We present the structure showing the molecular interactions of the TPR domain of AIP in complex with the peptide-binding motifs of HSP90 and TOMM20. Our results show that no known disease-associated mutation causes loss of binding of chaperones alone. However, a subset of mutations affects binding of client proteins to the Cα-7h of AIP. Consequently, loss of client protein interaction with the Cα-7h of AIP is sufficient for pituitary adenoma predisposition.

## Materials and Methods

### Protein Purification

The TPR domain (residues 166–330) of human AIP was expressed as a PreScission cleavable His-tagged protein from pTWO-E (pET-17b derived; personal communication, A. W. Oliver, Sussex University). The TPR domain was purified by talon-affinity chromatography (Clontech, Oxford, UK), then concentrated and desalted on a HIPrep 26/10 desalting column equilibrated in 20 mM Tris pH 7.5 containing 1 mM EDTA. The sample was then cleaved overnight with GST-tagged PreScision protease. The cleaved protein was subsequently passed through a GST column equilibrated in 20 mM Tris pH 7.5, 1 mM EDTA and 150 mM NaCl and then through a second Talon column to remove any remaining uncleaved protein. The flow through was then concentrated and subjected to superdex 75HR gel-filtration chromatography equilibrated in 20 mM Tris pH 7.5, 1 mM EDTA, 1 mM DTT and 500 mM NaCl. Pure TPR domain were concentrated and then desalted on a HIPrep 26/10 column equilibrated in 20 mM Tris pH 7.5 containing 1 mM EDTA. The protein was stored frozen at 2 mg ml^−1^.

### Structure Determination and Analysis

Human AIP TPR-domain was mixed with EDASRMEEVD (HSP90β) or AQSLAEDDVE (TOMM20) peptide at a 1∶20 molar ratio and concentrated to 15 mg ml^−1^. Crystals of AIP TPR-domain in complex with peptide were obtained at 7.5 mg ml^−1^ from sitting well drops equilibrated against 1 M ammonium sulphate, 1% PEG 3350, 0.1 M Bis-Tris pH5.5. Crystals appeared at 14°C and were harvested by successive transfer to crystallization buffer with increasing glycerol to 30%. Crystals were flash frozen in liquid nitrogen. Diffraction data were collected from crystals frozen at 100 K on Station I03 at the Diamond Light Source (Didcot, UK). Refinement was carried out using Phenix Refine [37], [38], and manual rebuilding was performed in Coot [39]. All other programs used were part of the CCP4 suite [40]. Evolutionary conservation was calculated using the ConSurf server [41]–[43] and conservation, as well as other PDB files, displayed using PyMol (The PyMOL Molecular Graphics System, Version 1.2r3pre, Schrödinger, LLC, USA).

### Isothermal Titration Calorimetry and HSP90 ATPase Assays

The heat of interaction was measured on an ITC_200_ microcalorimeter (Microcal), with a cell volume of 200 µL, under the same buffer conditions (20 mM Tris, pH 7.5, containing 5 mM NaCl) at 30°C. Twenty 1.9 µL aliquots of AIP TPR-domain at 350 µM were injected into 30 µM of human HSP90β. For peptide interactions twenty 1.9 µL aliquots of peptide ranging from 350 to 600 µM were injected into 30 µM of AIP TPR-domain. Heats of dilution were determined in a separate experiment by diluting protein or peptide into buffer, and the corrected data were fitted using a non-linear least-squares curve-fitting algorithm (Microcal Origin) with three floating variables: stoichiometry, binding constant and change in enthalpy of interaction. ATPase assays were previously described [44]–[46].

### Co-immunoprecipitation

The vectors used were pCI-neo-AIP-Flag and pcDNA 3.0-Myc-AIP, containing wild-type AIP cDNA with the Flag tag located downstream AIP and the Myc tag placed upstream, respectively. GH3 cells (3.6×10^6^) were cultured in Dulbeccós Modified Eaglés Medium (SIGMA) containing 10% fetal bovine serum and 1% penicillin/streptomycin (SIGMA) for 24 hours before transfection with 2.5 µg of each vector, using Lipofectamine 2000 (Life Technologies). The cells were then lysed (20 mM Tris-Cl pH 8.0, 200 mM NaCl, 1 mM EDTA pH 8.0, 0.5% Igepal and Complete Protease Inhibitor Cocktail [Roche]) and ∼150 µg of total protein was used for immunoprecipitation with 1 µg of TOMM20 peptide and 2 µg of either anti-Myc (SIGMA), anti-Flag (SIGMA) or mouse IgG (SIGMA) antibodies, respectively. Co-immunoprecipitation was carried out with Protein G Sepharose 4 Fast Flow (GE Healthcare) according to the protocol suggested by the manufacturer. Finally, the proteins were eluted by incubation for 5 minutes at 95°C with 40 µl of 1× Laemmli buffer, fractionated by SDS-PAGE and then transferred to a nitrocellulose membrane. Proteins were detected with 1∶3000 of either anti-Myc or anti-Flag antibodies. The bands were visualized on an Odyssey infrared scanner after incubation with of 1∶20000 goat anti-mouse 680 IRDye secondary antibody (Licor). As controls, we performed the same experiments using the following combinations of vectors: pCI-neo-Flag+pcDNA 3.0-Myc, pCI-neo-AIP-Flag+pcDNA 3.0-Myc, pCI-neo-Flag+pcDNA 3.0-Myc-AIP and no co-transfection.

## Results

### The Structural Features of the TPR Domain of AIP in Complex with HSP90 and TOMM20 Peptide

**Figure 2:**
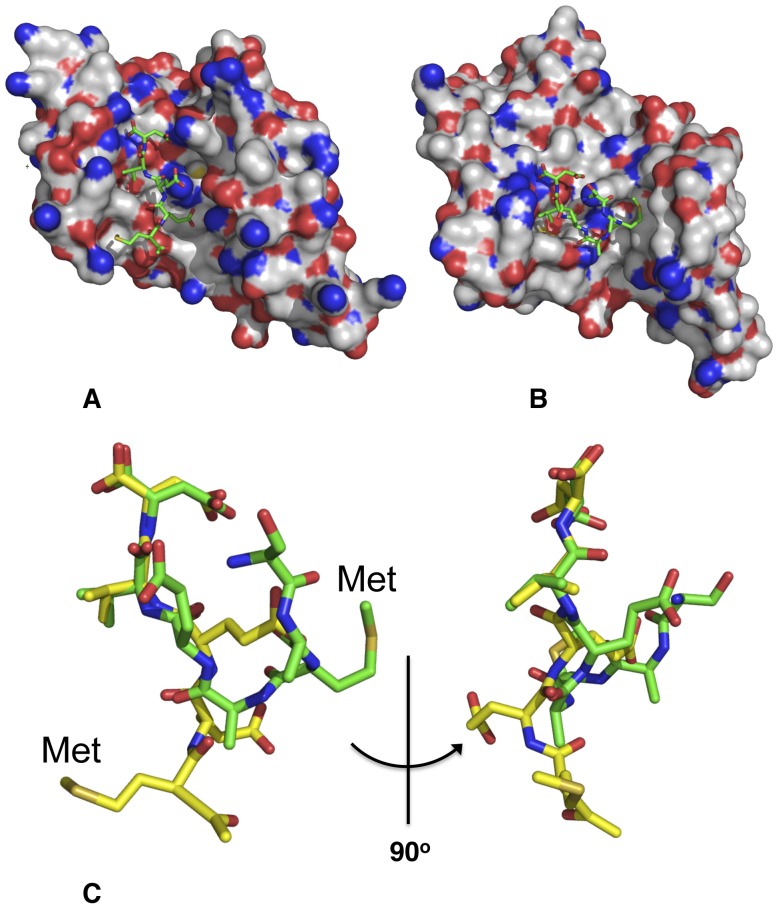
Binding of peptide to the TPR domains of Hop and AIP. (A), PyMol Space-filling model showing the binding of the MEEVD peptide of HSP90 to the TPR domain of Hop TPR2A and (B), the EDASRMEEVD peptide of HSP90β bound to the TPR domain of AIP (only SRMEEVD of the peptide is shown). (C), Superimposition of the peptides bound to the TPR domains of HOP2A (yellow) and AIP (green).

TPR domains that bind HSP90, HSP70 and TOMM20 are known to bind a specific short conserved motif at the C-terminal end of these chaperones (HSP90, MEEVD; HSP70, IEEVD; and TOMM20, EDDVE) [11], [12], [47]. The structures of the AIP TPR-domain in complex with peptide fragments from human HSP90β and TOMM20 were solved at 2.0 (PDB 4AIF) and 1.9 Å (PDB 4APO) resolution, respectively (Table 1). The TPR domain of AIP is similar to other TPR-domain proteins consisting of three pairs of anti-parallel helices and a Cα-7h (Fig. 1A). We found that the EDASRMEEVD (HSP90) and AQSLAEDDVE (TOMM20) peptides bind within the TPR-domain cleft and adopt a similar backbone conformation (Fig. 1). The mode of interaction of these peptides resembles that of the HSP90 and HSP70 C-terminal peptides binding to the TPR-domain of CHIP rather than that of HOP [11], [12] (Fig. 1 and 2).

**Table 1:** Crystallography statistics.

Data collection	HSP90 (SRMEEVD)*	TOMM20 (AEDDVE)*
Space group	C2	C2
Unit cell a, b, c (Å)α β, γ (°)	63.82, 104.49, 69.2790, 97.41, 90	60.2, 106.82, 68.4790, 100.85, 90
Maximal resolution (Å)	2.01	1.9
Highest resolution bin	2.06−2.01	2−1.9
Observations	98171	92304
Unique reflections	29974	28523
Completeness (%)	99.4(98.5)	84.8 (68.1)
Rmerge	0.061(0.588)	0.049 (0.246)
Mean I/σI	10.9(2.4)	13.6 (4.2)
Multiplicity	3.3(3.2)	3.2 (3.1)
**Refinement**	**HSP90 (SRMEEVD)** *	**TOMM20 (AEDDVE)** *
Total atoms	2732	3007
Protein atoms	2402	2486
Ligand atoms	100	94
Residues modeled	D/1-7;E1-7	D/1-6; E/1-6
Non-protein residues modeled	327 waters, 1SO_4_	484 waters, 1 SO_4_, 1 PEG
Resolution range (Å)	40.4−2.01	28.46−1.9
Rconv	0.1879	0.1809
Rfree	0.236	0.2344
Residues in most favored regions (%)	98	100
Residues in allowed regions (%)	99.7	100
Residues in outlier regions (%)	0.3	0
RMSD bond (Å)	0.006	0.006
RMSD angle	0.960	0.906
Mean B-factor (Å^2^)	Protein 36.03Solvent 50.34	Protein 29.98Solvent 45.1

* 10-mer peptides were used in the crystallization, but only 6–7 resides were visible.

**Figure 3:**
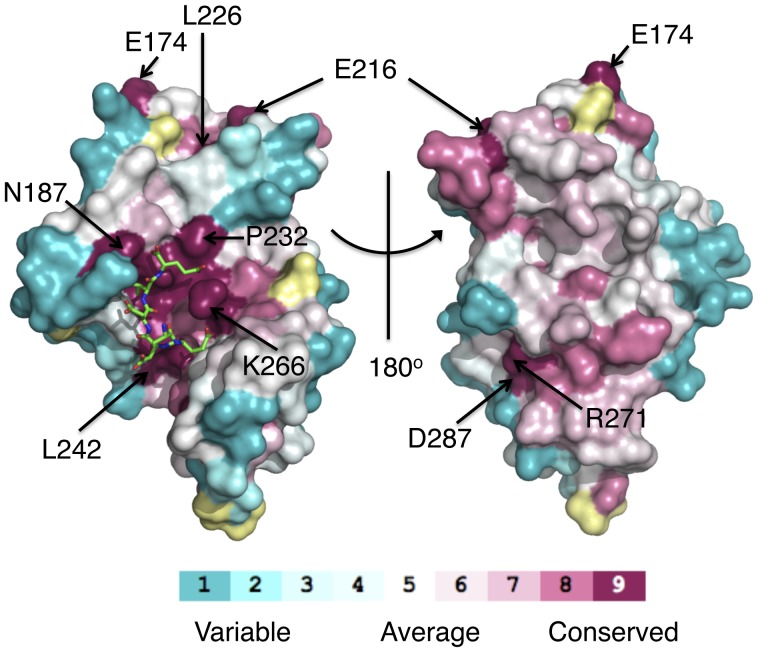
PyMol diagram showing the conservation of residues on the surface of AIP TPR-domain. The most highly conserved residues line the cavity of the TPR domain in which the TPR-motif containing peptides bind to.

**Figure 4:**
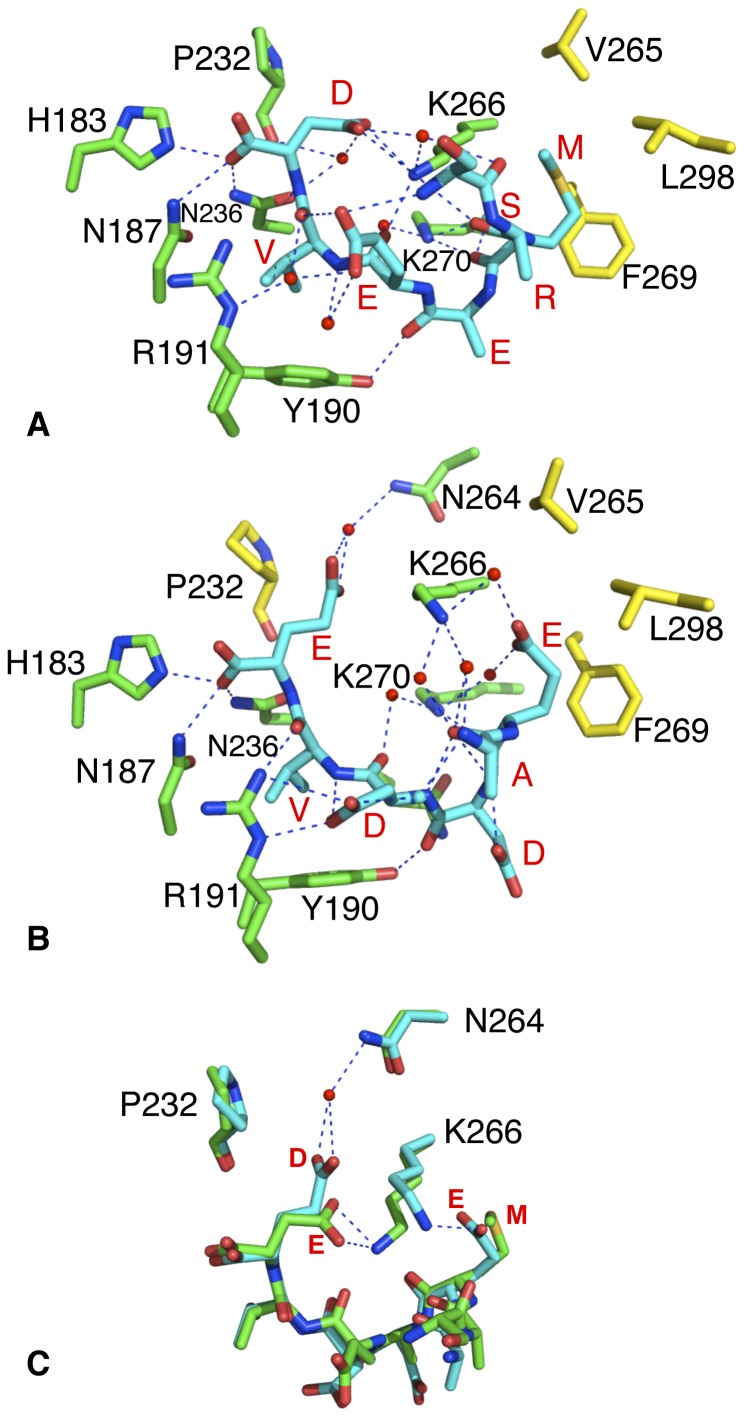
PyMOL diagram showing binding interactions. (A) Interactions with HSP90β EDASRMEEVD peptide and (B), with TOMM20 AQSLAEDDVE peptide bound to the TPR domain of AIP. Peptide residues that where visible (SRMEEVD and AEDDVE) are shown in red as single letter code. Dotted blue lines represent hydrogen bonds and green, the amino acid residues involved; red-colored spheres, water molecules and yellow residues, residues solely in van der Waals contact. The structures were obtained at 2.0 (PDB, 4AIF) and 1.9 Å (PDB, 4APO), respectively. (C), Molecular switching in the TPR domain of AIP. The alternative conformations of Lys 266 allow selection of the Hsp90 MEEVD- (green) or TOMM20 EDDVE-motif (cyan). Dotted blue lines represent hydrogen bonds while red-colored spheres represent water molecules.

The residues lining the TPR-binding site are highly conserved (Fig. 3). The structures show that the C-terminal carboxylate group and the C-terminal aspartate (HSP90) or glutamate (TOMM20) side-chain are involved in a series of hydrogen bonds that is reminiscent of the carboxylate clamp seen in the MEEVD-HOP complex [11] (Fig. 4). In the AIP-EDASRMEEVD (HSP90) structure the C-terminal carboxylic acid makes direct hydrogen bonds to one of the ring nitrogens of His 183 and to the amine nitrogen of Asn 187 and Asn 236. The aspartate group makes water-mediated interactions to the main-chain carbonyl of Pro 232, to the carboxylic-acid oxygen of Asn 236, to the amine-group nitrogen of Lys 266 as well as an intramolecular interaction to the main-chain carbonyl of the serine of the EDASRMEEVD peptide. The C-terminal aspartic acid side-chain carboxyl-group also forms a direct interaction with the amine group of Lys 266.

For the peptide valine the main-chain carbonyl is hydrogen bonded to the secondary amine of Arg 191 via a water molecule, and via this same water molecule but also directly, to the carboxylic-acid oxygens of the second glutamate residue of the HSP90 peptide (EDASRMEEVD). The other carboxyl-group oxygen of this glutamate (EDASRMEEVD) is hydrogen-bonded to the secondary amine of Arg 191, while the other oxygen forms an intramolecular interaction with the main-chain amide of serine of the HSP90 peptide. The main-chain amide group of the peptide valine is also hydrogen bonded to one of the oxygens of the second glutamate of the peptide. The valine side-chain is itself packed into a hydrophobic pocket formed by the side chains of Asn 187, Tyr 190, Arg 191 and Asn 236.

The carbonyl of the second glutamate of the HSP90 peptide (EDASRMEEVD) forms a direct interaction with the side-chain amine of Lys 266, and via a water molecule to the side-chain amine group of Lys 270. The side-chain hydroxyl of Tyr 190 forms a direct interaction with the main-chain carbonyl of the second glutamate in the HSP90 peptide.

The methionine of the HSP90 peptide is itself packed into a hydrophobic pocket formed by the side chains of Val 265, Lys 266, Phe 269, Lys 270 and Leu 298. Interestingly, Lys 266 was predicted to be a ligand-binding residue [48]. However, the main-chain amine of the peptide methionine also forms both a direct interaction and a water-mediated hydrogen bond with the side-chain amine of Lys 270. The main-chain carbonyl of the peptide arginine directly interacts with the side-chain amine of Lys 266, while the main-chain carbonyl of the peptide serine forms hydrogen bonds via a water molecule to the side-chain amine of Lys 266.

Interactions between the TOMM20 peptide and the TPR domain of AIP are similar but not identical. The main differences result due to the need to pack the first glutamate side-chain of the TOMM20 peptide (AQSLAEDDVE) into the hydrophobic pocket that accepts the methionine residue in the case of HSP90 peptide (MEEVD) (Fig. 4). While the side chain of this glutamate enters the hydrophobic pocket the carboxylate oxygens point back towards and interacts with the side-chain amine of Lys 266. Consequently, Lys 266 adopts an alternative conformation to that seen with the HSP90 bound peptide. The conformational change in Lys 266 acts like a switch that not only allows the binding of the TOMM20 glutamate in the methionine pocket, but also allows the longer C-terminal glutamate side-chain of TOMM20 (Asp in HSP90), to pack between the side chain of Pro 232 and Lys 266; and consequently form a hydrogen bond via a water molecule with the side-chain amine of Asn 264 (Fig. 4C).

Although attempts to obtain the structure of an equivalent HSP70 peptide bound to the TPR domain of AIP failed, we assume that the isoleucine (IEEVD) binds to the same hydrophobic pocket as the methionine of HSP90.

### Dimerization of the AIP TPR-domain and the Role of Arg 304

**Figure 5:**
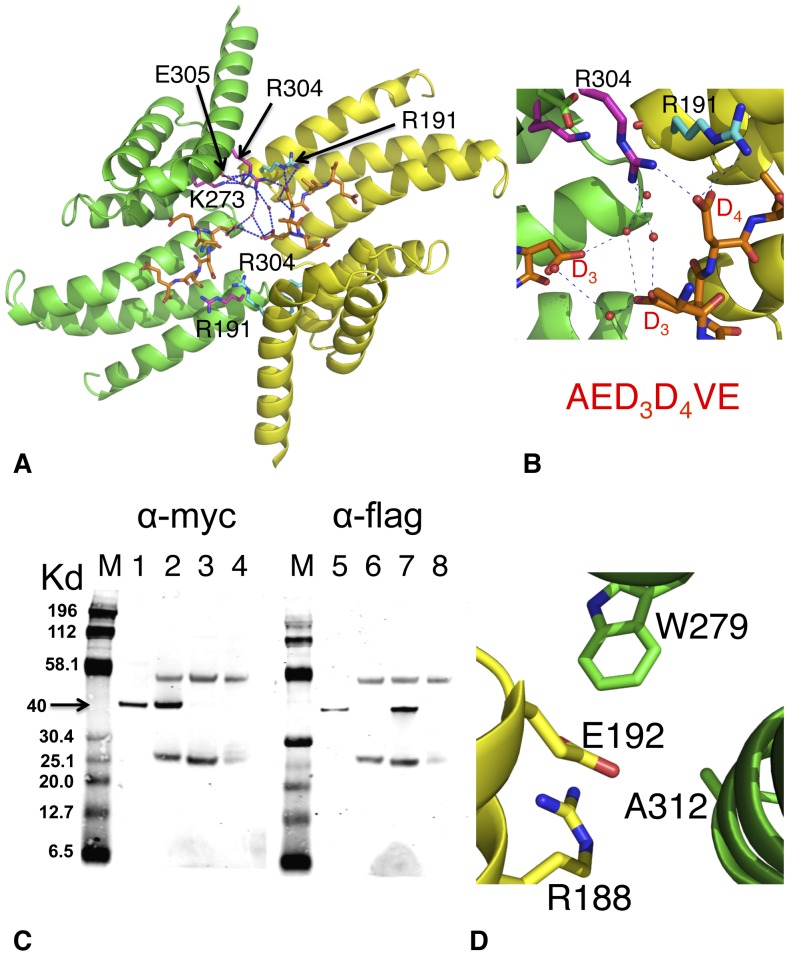
PyMol cartoon showing dimerization of AIP TPR-domain through crystal lattice contacts. (A), The AIP domains are in green and yellow. Amino acid residues are in magenta or cyan, hydrogen bonds as blue dotted lines, water molecules as red spheres and bound TOMM20 AQSLAEDDVE-peptide used in the crystallization in gold. However, only residues AEDDVE are visible in the structure. The TPR domains are symmetrically related and hydrogen bonding is shown in only one half of the figure. The cartoon shows that Arg 304 is hydrogen bounded directly to the neighboring TOMM20 bound peptide (gold). (B), PyMol cartoon showing a close up of the main interactions between Arg 304 and bound peptide used in the crystallization (AQLSLAED_3_D_4_VE) in panel A. However, only residues AED_3_D_4_VE are visible in the structure. (C), Co-immunoprecipitation of Flag-AIP and Myc-AIP in the presence of TOMM20 peptide (AQSLAEDDVE). The results show that Flag-AIP and Myc-AIP do not co-immunoprecipitate. M, molecular mass markers, with molecular mass indicated to the left of the panel; lane 1 and 5 AIP input (cleared lysate) protein; lane 2 and 6 are anti-Myc co-immunoprecipitation, lanes 3 and 7 are anti-Flag co-immunoprecipitations, while lanes 4 and 8 are IgG control. Lanes 1–4 (first gel) was blotted for Myc tag and lanes 5–8 (second gel) for Flag tag. The arrow indicates the position where the flag- and myc-tagged AIP runs (40 Kd). (D), The core interaction of the AIP dimerization interface shows that E192 is buried and shielded from solvent by Ala 312, Arg 188 and Trp 279.

The crystal structure of the TPR domain of AIP in complex with peptide revealed the possibility that the TPR domain might form a biological dimer (Fig. 5A and B). Significantly, Arg 304, whose missense mutation is linked to disease, was found to form interactions with the aspartates of the TOMM20 peptide (AQSLAEDDVE) bound in the neighbouring TPR domain. In contrast, in the HSP90 peptide bound structure Arg 304 is disordered and no significant interactions are made. The question therefore arises as to whether Arg 304 is naturally involved in intermolecular or intramolecular interactions with bound peptide (or intact chaperone) and whether the TPR AIP-domain forms a biological dimer. It has been previously reported that two molecules of AIP can be found in some HSP90 complexes, but whether AIP was a biological dimer in these complexes was not established [49].

We conducted reciprocal co-immunoprecipitations experiments (Fig. 5C) in which cells were transfected with Flag- and Myc-tagged AIP in the presence of the TOMM20 peptide. We found that using either anti-Flag or anti-Myc antibodies failed to co-immunoprecipitate the tagged proteins suggesting that AIP dimerization is not biologically relevant. Furthermore, using isothermal titration calorimetry (ITC) the stoichiometry of the interaction between the TPR domain of AIP and HSP90β was found to be 0.3∶1, showing that one molecule of AIP interacts with a dimer of HSP90 (Table 2). In addition, the E192R AIP mutant, where Glu 192 forms the core of the interaction interface of the AIP dimer (Fig. 5D), did not alter the stoichiometry or thermodynamics of the interaction with HSP90β (0.47∶1, AIP-TPR-E192R: HSP90β, Table 2). We next tested the effect of mutating Arg 304, which directly interacts with TOMM20 peptide bound in the neighbouring AIP molecule. The binding of HSP90β, HSP70 and TOMM20 peptides to the R304A and R304Q mutants was unaltered relative to the wild type interaction (Table 2). Failure to form stable dimerization of AIP, caused by these mutations, would significantly change the thermodynamic properties of the interaction. We therefore conclude that the dimerization interface seen in the crystals is not a true biological interface, but a crystallographic one.

**Table 2:** Isothermal titration calorimetry binding of AIP and target.

TPR-domain	Ligand	Kd (µM)	N	ΔH(cal/mol)	ΔS(Cal/mol/deg)
WT	FL-hHSP90β	13.3±1.8	0.30	−4554	7.29
E192R	FL-hHSP90β	11.1±1.3	0.47	−4114	9.11
WT	MEEVD (HSP90)	12.6±1.6	1.0	−4140	8.75
WT	EDASRMEEVD (hHSP90β)	18.6±2.014.4±1.0	1.21.1	−5041−3921	5.029.22
R304A	EDASRMEEVD (hHSP90β)	15.6±0.85	0.96	−6435	0.76
R304Q	EDASRMEEVD (hHSP90β)	16.2±1.1	0.98	−6417	0.76
WT	DDTSRMEEVD (hHSP90α)	9.5±0.6	1.2	−3529	11.3
WT	GSGPTIEEVD (hHSP70)	18.1±1.9	0.63	−6348	0.76
R304A	GSGPTIEEVD (hHSP70)	22.8±1.6	0.66	−5456	3.24
R304Q	GSGPTIEEVD (hHSP70)	31.1±2.6	0.84	−5498	2.48
WT	AQSLAEDDVE (hTomm20)	12.3±0.5	0.87	−6765	0.16
R304A	AQSLAEDDVE (hTomm20)	16.6±0.5	0.69	−4508	7.0
R304Q	AQSLAEDDVE (hTomm20)	22.5±2.2	0.66	−6073	1.23
WT	TLEELDW (hPDE4A5)	64.5±3.2	0.89	−4418	4.6

### Selectivity in the Binding of Proteins to the TPR Domain of AIP

We next wanted to understand the selectivity for the different chaperones that bind to AIP and utilised ITC to measure the affinity for these interactions. The TPR domain of AIP bound full-length HSP90β with a *K*_d_ = 13.3±1.8 µM and showed a favourable entropic contribution (Table 2). The peptides representing the extreme C-terminus of HSP90 (MEEVD), HSP90β (EDASRMEEVD), HSP90α (DDTSRMEEVD) and TOMM20 (AQSLAEDDVE) also bound with similar affinities (*K*_d_ = 12.6±1.6; 14.4±1.0; 9.5±0.6 and 12.3±0.5 µM, respectively; Table 2), suggesting that the core interaction between these chaperones and the TPR domain of AIP involves the terminal five amino acids of these proteins.

Although we were unable to test the binding of intact PDE4A5 to AIP, we instead measured the affinity for the interaction of the peptide TLEELDW, which contains the core binding sequence LEELD (identified in PDE2A as LYDLD). The LEELD motif of PDE4A5 is not a C-terminal sequence and its ability to bind to the TPR domain is questionable. The binding affinity of the PDE4A5 peptide (TLEELDW; *K*_d_ = 64.5±3.2 µM) was found to be significantly weaker than the equivalent peptides form HSP90α, HSP90β and TOMM20 (*K*_d_ = 9.5, 14.4–18.6; and 12.3 µM, respectively; Table 2). Furthermore, a structural analysis of the PDE2A homologue indicates that the homologous LYDLD sequence is unlikely to be accessible for binding to the TPR domain of AIP as it is involved in folding of the protein. Consequently, the interaction of AIP with PDE4A5 is not mediated by binding to the LEELD peptide sequence.

### AIP does not Affect the HSP90 ATPase Activity

The first TPR-domain protein shown to influence the ATPase activity of HSP90 was Sti1p [45]. We wanted to see if AIP could similarly affect HSP90 ATPase activity. A 20-fold molar excess of full-length AIP did not influence the ATPase activity of HSP90 (Link to Supporting information).

### Disease Associated Mutations of AIP

Nonsense, splice variant and frameshift mutations (Table 3) clearly disrupt the TPR-domain of AIP and lead to a dysfunctional protein. However, the effect of missense mutations is difficult to predict. We have used our structure to define mutations associated with disease to understand how they might affect the function of this domain. Many of the missense mutations are involved in the folding and stability of the TPR AIP-domain. C238Y, K241E, I257V, R271W, and possibly A299V all disrupt either hydrophobic or polar interactions that impact on the folding of the domain (Table 3). In fact, attempts to purify C238Y and A299V resulted in much of the protein aggregating suggesting that the proteins were at least partly unfolded. In contrast, R304* (nonsense mutation), R304Q, Q307* and R325Q were identified as ‘disease-associated’ mutations that, *in vitro* at least, do not disrupt chaperone binding. Our ITC results show that for R304A and R304Q the HSP90, HSP70 and TOMM20 peptides bind normally (Table 2). Structural analysis showed that the Gln 307 and Arg 325 amino acid residues (all clearly visible in the TOMM20-AIP structure), are further away from the TPR domain-binding site than Arg 304, and are not involved in packing interactions or in binding of the bound conserved peptide motifs. Thus, at least *in vitro*, these residues do not disrupt chaperone binding although they have been strongly implicated in causing FIPA [4], [6], [50]–[52]. Furthermore, the extreme C-terminus of AIP has been shown to represent the binding site for the client proteins AhR and PDE4A5 [48], [52]. These results suggest that disruption of client-protein binding alone is sufficient for pituitary tumor predisposition.

**Table 3:** Classification of the effect TPR-AIP mutations on its structure.

Mutation	Mutation type	AIP domain	Probable effect of the mutation
Q184*	Nonsense	TPR domain	Non-functional
K201*	Nonsense	TPR domain	Non-functional
E216*	Nonsense	TPR domain	Non-functional
Q217*	Nonsense	TPR domain	Non-functional
E222*	Nonsense	TPR domain	Non-functional
C238Y	Missense	TPR domain	Disrupts packing of hydrophobic core
Q239*	Nonsense	TPR domain	Non-functional
C240R	Missense	TPR domain	Disrupts packing of hydrophobic core
K241E	Missense	TPR domain	Disrupts hydrogen bonding to Glu246
K241*	Nonsense	TPR domain	Non-functional
I257V	Missense	TPR domain	Disrupts packing of hydrophobic core
Y261*	Nonsense	TPR domain	Ligand binding
K266A	Missense	TPR domain	Ligand binding
Y268C	Missense	TPR domain	Disrupts packing of hydrophobic core
Y268*	Nonsense	TPR domain	Non-functional
R271W	Missense	TPR domain	Disrupts hydrogen bonding to Asp287 and Ser255
A277P	Missense	TPR domain	Disrupts hydrophobic packing against Tyr 247
A291M/E	Missense	TPR domain	Disrupts packing of hydrophobic core. (Forms base of hydrophobic pocket interacting with bound peptide)
A299V	Missense	TPR domain	At start of Cα-7h and may disrupt some small degree of packing with Leu292
R304*	Nonsense	TPR domain	Weakens PDE4A5 binding (see E304Q and [52]) and would disrupt AhR binding
R304Q	Missense	TPR domain	Weakens PDE4A5 binding (see [52])
Q307*	Nonsense	TPR domain	Would disrupt AhR binding
R325Q	Missense	TPR domain	Potentially client-protein binding. One residue short of the 5-residue deletion that disrupts AhR binding

**Figure 6:**
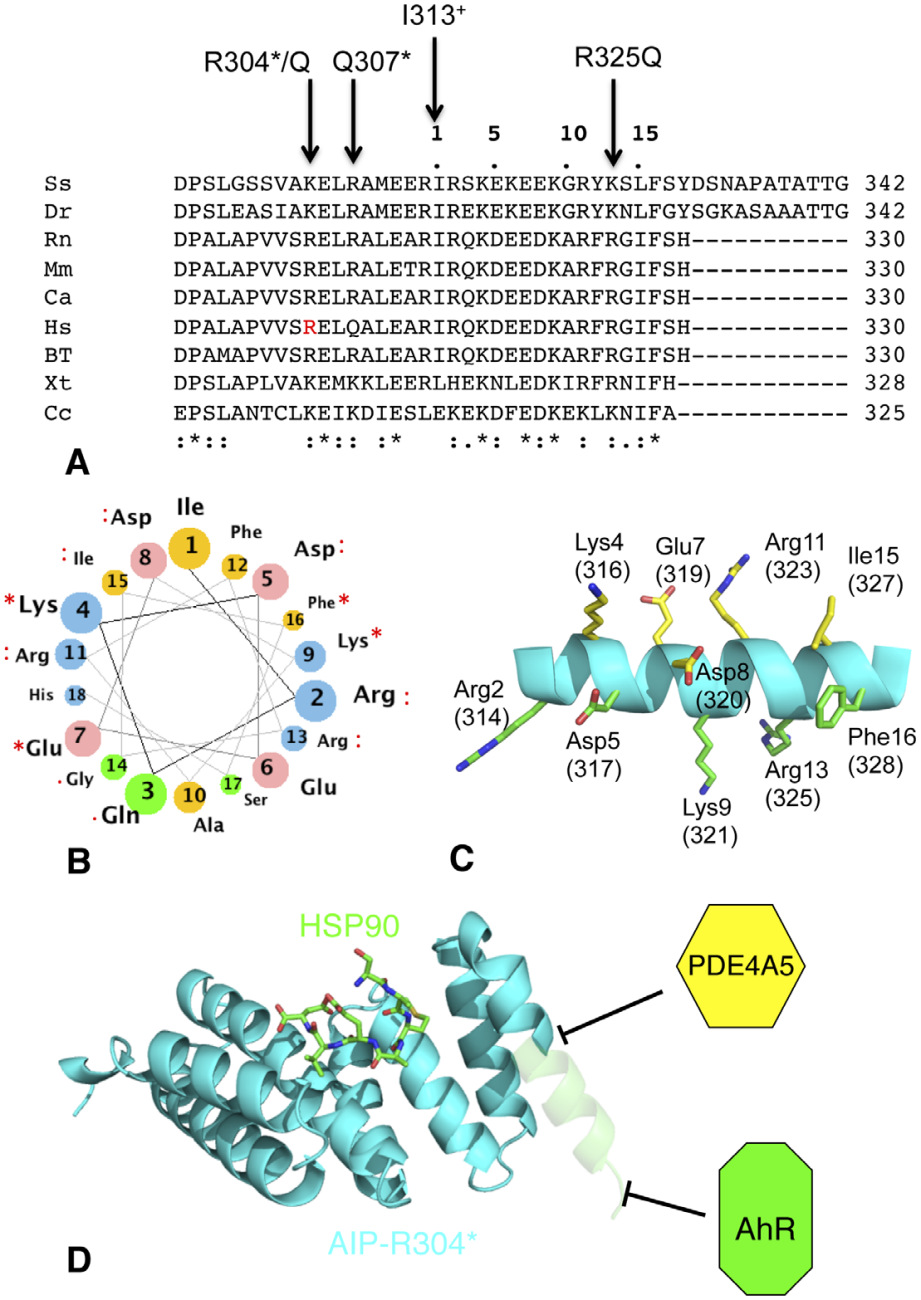
Sequence conservation of the Cα-7h of AIP. (A), sequence alignment showing conservation of amino acid residues. Ss, Salmo salar (NM_001140060.1); Dr, Danio rerio (NM_214712.1); Rn, Rattus norvegicus (NM_172327.2); Mm, Macaca mulatta (NM_001194313); Ca, Chlorocebus aethiops (O97628); Hs, Homo sapiens (FJ514478.1); Bt, Bos taurus (NM_183082.1), Xt, Xenopus (Silurana) tropicalis (NM_001102749.1) and Cc, Caligus clemensi (BT080130.1). (I313^+^), Ile 313 represents the last residue in the sequence that is involved in packing interactions of the TPR domain. Mutations associated with disease are indicated above the sequence. (* below the sequence), Amino acids at these positions are identical; (:), highly conserved (.) or conserved. Arg 304 of Human AIP is shown in red type face. Numbers above the sequence (positions 1 to 15) represent residue numbers of the helical wheel shown in panel B. (B), Helical wheel showing the position of identical and conserved residues form the alignment in panel A for the Cα-7h of AIP. Orange, non-polar; green, polar uncharged; pink, acidic and blue, basic amino-acid residues. (C), PyMol cartoon showing a hypothetical helix (residues beyond Arg 325) with the identical and highly conserved amino acid residues shown in panels A and B. Conserved residues on one side of the helix are shown in green and on the other in yellow. Residue numbers shown are those in panel B, while those in brackets are actual residue numbers in panel A. (D), The TPR-domain of the R304* mutant of AIP. Deletion of the terminal region of AIP (transparent helical region) allows chaperone binding but disrupts association with PDE4A5 and AhR.

Further analyses of the extreme C-terminus of the Cα-7h shows that there are a number of conserved charged and hydrophobic residues (Fig. 6). These residues are predicted to be part of a helical structure (PSIPRED, UCL Department of Computer Science, Bioinformatics Group), and form two conserved regions on either side of the helix (Fig. 6C). Hydrophobic residues beyond Ile 313, the last residue in the structure that is involved in packing with the main fold of the domain, would not be buried if the Cα-7h continues as such into solvent. The conservation of these residues suggests that they represent a binding site for specific client proteins; especially for AhR and PDE4A5, which are client proteins known to interact with this helix (Fig. 6D).

## Discussion

The structure of the TPR domain of AIP in complex with peptide representing the TPR-domain binding motif from HSP90 and TOMM20 were determined to high resolution. The structure with TOMM20 peptide showed that electron density for residues Asp 172 to Arg 325 of the TPR domain was visible. We show that HSP90, HSP70 and TOMM20, but not the PDE4A5 TLEELDW-peptide, can interact with the TPR domain of AIP with similar affinity. Using ITC we show that the stoichiometry of the interaction between AIP and intact HSP90 was 0.5∶1 (AIP:HSP90).

AIP binding of these conserved peptide sequences is similar to that observed for CHIP, rather than that seen with HOP. Unlike HOP-bound peptides, for AIP and CHIP the upstream sequence of the peptides is directed up and out of the binding cleft to avoid interaction with these upstream sequences, which differ between HSP70 and HSP90 [11], [12]. HOP is a co-chaperone of HSP90 that not only acts as scaffold between HSP70 and HSP90, but also silences the ATPase activity of HSP90 [45]. It thus stalls the ATPase-coupled conformational cycle of HSP90 and allows client protein loading from HSP70 to HSP90 [11], [44], [45]. HOP binds HSP70 and HSP90 using separate TPR-domain modules [11], and therefore can associate with both chaperone systems simultaneously. CHIP on the other hand is a U box E3 ubiquitin ligase that binds either HSP70 or HSP90 using a single TPR-domain module and appears to ubiquitinate client proteins of these chaperone systems [12], [53], [54]. The conformation that the bound peptides adopt with HOP and CHIP/AIP is largely dependent on the position of the hydrophobic pockets that accept the methionine and valine amino acid residues of the conserved binding motif MEEVD, in the case for HSP90. Thus, the TPR domains can be reclassified depending on the relative position of these hydrophobic pockets. When both pockets are on the same side of the TPR-binding cleft we observe the HOP-type mediated binding (*cis*-mode). When the methionine pocket is on the other side we see the CHIP/AIP (*trans-*mode) of binding. The *trans*-mode of binding appears to be used where numerous similarly related peptides are binding to the same TPR domain.

Another interesting feature by which AIP accommodates these different TPR-binding sequences is by way of a specific side chain rearrangement (Fig. 4C). The methionine side-chain of the conserved MEEVD motif being hydrophobic in nature can enter the appropriate hydrophobic pocket, while for the TOMM20 glutamate its side chain enters the hydrophobic pocket but the carboxylic acid group points back out and interacts with the side-chain amine group of Lys 266. The ‘switched’ conformation of Lys 266 then allows the side-chain of the C-terminal glutamate to pack between Lys 266 and Pro 232 and to form a water-mediated interaction to the side-chain amine of Asn 264. In contrast, for the HSP90 peptide, Lys 266 forms direct hydrogen bonds with the carboxyl group of the shorter C-terminal aspartic acid side-chain as part of the carboxylate clamp. Thus, the rearrangement that allows the glutamate residue of TOMM20 (EDDVE) to bind the hydrophobic pocket also allows the longer C-terminal glutamate side-chain of TOMM20 to be accommodated.

Analysis of the mutations that occur in AIP in the context of the structure (residues visible were Asp 172 to Arg 325) has allowed us to define their effects on the structural integrity of the AIP protein. Most mutations affect the structural integrity of the TPR domain (Table 3). However, no single mutation of the TPR domain prevents chaperone binding alone. In contrast, a subset of disease-associated mutations of conserved residues of the Cα-7h that affect client-protein binding alone was identified. Our structures and ITC data show that for the R304A/Q mutations chaperone binding is unaffected. For Gln 307 and Arg 325 these residues are further away from the TPR domain binding-site and are not involved in domain packing interactions. Consequently, they are not part of the chaperone-binding site of the TPR-domain. Interestingly, the R325Q mutation is one residue short of the 5-residue deletion that disrupts AhR binding [5], [48], [52]. Of these five residues only two are conserved, Ile 327 and Phe 328 (Fig. 6), which alone are unlikely to represent the complete interaction site of AhR as this would be very weak. Consequently, the extensive conservation of the Cα-7h is likely to represent an interaction site for at least AhR. However, the R304Q mutation is also known to slightly destabilize the PDE4A5 interaction [52], providing clear evidence that the conservation in the Cα-7h represents a binding site for client proteins (Fig. 6D). Our results therefore suggest that the primary change in a subset of AIP mutant of the Cα-7h is loss of association with, at least some, client proteins. Whether *in vivo* this also leads to a breakdown in the association of HSP90 and AIP is currently unknown and warrants further investigation. However, it appears that AIP acts as a co-chaperone that delivers client protein to HSP90, in common with other co-chaperones of HSP90 such as HOP and CDC37 [14].

**Figure 7:**
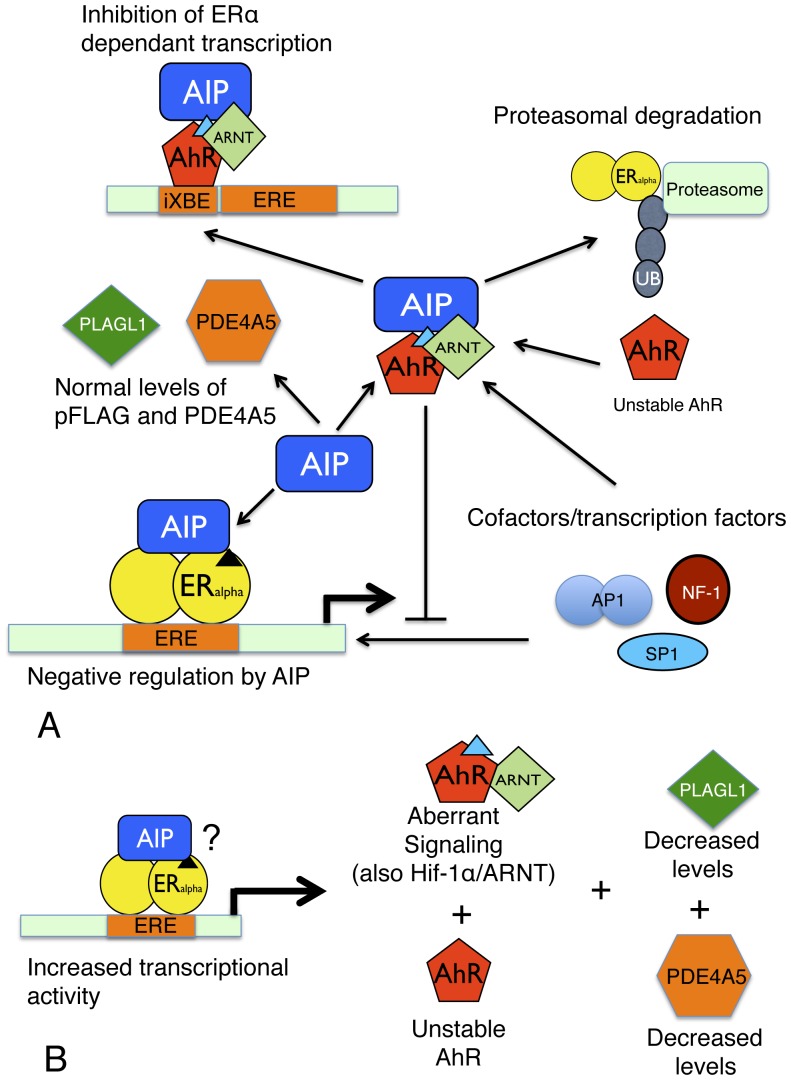
Model showing the affect of mutant AIP on cellular signaling pathways. (A), Wild type AIP stabilizes AhR, which in turn downregulates ERα dependent transcription by promoting the ubiquitination and proteasomal destruction of ERα, by competing for specific cofactors required for ERα dependent transcription and by binding to iXRE sites that block ERα dependent transcription. AIP downregulates transcription by ERα at ERE sites. AIP is also known to maintain cellular levels of PLAGL1 and PDE4A5. Small triangles represent ligand bound to their appropriate receptor. (B), Mutant AIP fails to bind AhR, PDE4A5 and possibly ERα, resulting in unstable AhR and PDE4A5 and perhaps upregulation of transcription at ERE sites. A decline in levels of PLAGL1 and changes in cAMP concentration also result. The question mark emphasizes that AIP may or may not interact with ERα at ERE sites, but if it does it may fail to provide appropriate negative regulation.

The destabilization of AhR would naturally imbalance assembly of AhR/ARNT complex and it has been shown that levels of either ARNT or ARNT2, but not both, are devoid in AIP-deficient mouse pituitary tumors [55]. Furthermore, PDE2A, which is AIP dependent, inhibits nuclear translocation of AhR by lowering cAMP levels. Consequently, elevated and aberrant cAMP signalling, often seen in pituitary tumors, may imbalance AhR/ARNT and ARNT/Hif-1e signalling. Disruption of AhR binding to AIP might also have profound effects on ERα-dependent transcription. Loss of AIP binding to AhR causes degradation of AhR [56]. Thus, a model can be proposed (Fig. 7), which results in the destabilization of AhR, which could then upregulate expression from ERα dependant promoters by affecting several different mechanisms. Thus, AhR would not compete for ERα cofactors and transcription factors, would fail to promote the proteasomal degradation of ERα and would not be available for binding to inhibitory xenobiotic response elements (iXRE), that downregulate specific ERα-directed expression [57]. However, the exact effects on ERα levels and ERα-directed transcription are currently unknown. Certainly work by Cai *et al*., [58] shows that AIP acts as a negative regular of ERα. Although, the same authors show that ERα is still able to associate with AIP R304* mutant. Furthermore, these experiments used overexpressed AIP mutant and therefore do not address whether the mutation fails to provide the normal negative regulatory effect on ERα under normal AIP levels. Interestingly, other mutations, such as Q217*, that disrupt the TPR domain were seen to activate ERα directed transcriptional activity. Furthermore, it is also known that tumor suppressor levels of PLAGL1 decline in the absence of functional AIP, but the mechanism leading to this is poorly understood. None-the-less the loss of an important tumor suppressor is likely to have some role in the formation of pituitary adenomas. However, it is evident that because of AIP’s promiscuity a variety of biochemical changes in pituitary cells occur under conditions when AIP is non functional. Consequently, pituitary tumor predisposition is likely to be a result of these biochemical changes that may all contribute to a varying degree in the process.

In conclusion, our results show that Arg 304 does not play any significant role in mediating AIP binding to HSP90, HSP70 or TOMM20. Taken together with the highly conserved C-terminus of AIP, and specific mutations that occur on the Cα-7h, our results support the idea that this helix is involved in client protein interactions, at least with AhR and PDE4A5, and that loss of such interactions leads to a variety of biochemical changes in pituitary cells that predisposes to pituitary adenoma. Consequently, understanding the role AIP plays in maintaining and activating Cα-7h interacting client proteins will help towards understanding the cellular events that lead to pituitary tumor predisposition. This study forms the springboard for more detailed investigations in isolating AIP client-proteins that when deregulated predispose to pituitary tumors.

## Supporting Information

Figure S1 (TIF)
