# Supplementary figures and images for: Structure of the TPR Domain of AIP: Lack of Client Protein Interaction with the C-Terminal α-7 Helix of the TPR Domain of AIP Is Sufficient for Pituitary Adenoma Predisposition
Source: PLoS One. 2012 Dec 31;7(12):e53339. doi: 10.1371/journal.pone.0053339 (PMC3534021; doi:10.1371/journal.pone.0053339)

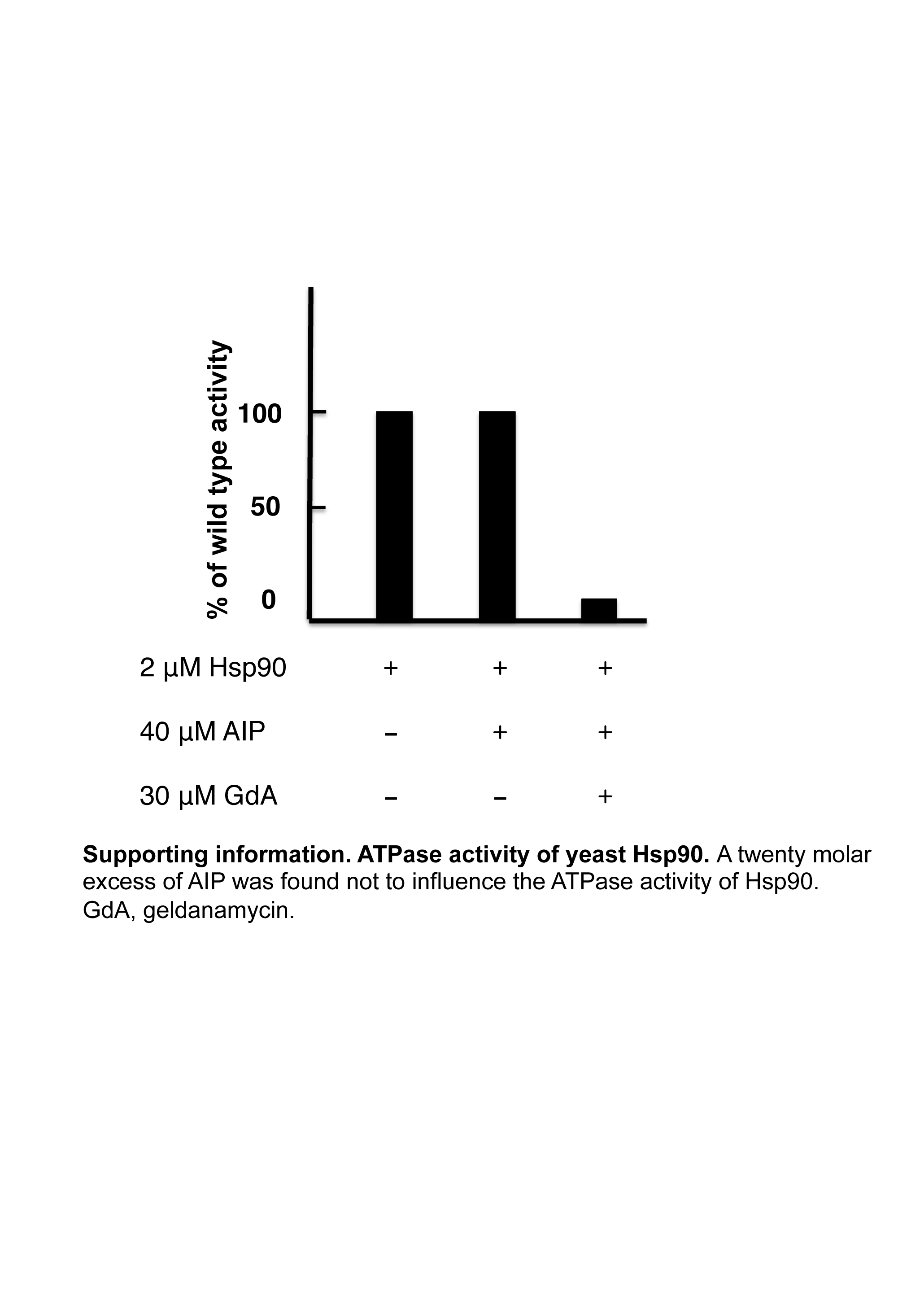

Supplement: Figure S1 — (TIF) [file pone.0053339.s001.tif]
